# On the Re-establishment of Nervous Action after Autoplastic Operations

**Published:** 1845-06

**Authors:** 


*On the Re-establishment of Nervous Action after Autoplastic Operations.*—M. Jobert (de Lamballe) has arrived at the following results, through his experiments on man and on animals:—1st. Immediately after autoplastic operations, sensibility diminishes or disappears in the flaps, the diminution of the sensibility being directly in reason of the loss of blood. 2d. The section of the pedicle is followed by complete loss of sensibility, but after a certain length of time it reappears, and increases in the same proportion as the vascularity; both sometimes increase beyond the limits of the normal state, but always simultaneously. The anatomical examination of the pedicle gives the following results:—1st. After its section, the flaps are separated from all parts of the economy by cicatricial tissue. 2d. The only means of communication that exist between the flaps and the rest of the economy, are the vessels, more or less developed, which pass through the cicatricial tissue; no nervous filaments are ever met with. 3d. The nerves which existed primitively in the flap become atrophied, and may end by disappearing. Those of the neighbouring parts stop at the level of the cicatrix. Sometimes they are suddenly interrupted, presenting a kind of swelling at the neurilemma; sometimes they lose themselves in the cicatricial tissue, but it is never possible to follow them into the flap.

